# Clinicians’ views on a patient decision aid for deep brain stimulation in Parkinson’s disease

**DOI:** 10.3389/fnhum.2026.1851826

**Published:** 2026-07-13

**Authors:** Hillary S. King, Jennifer Blumenthal-Barby, Benjamin H. Levi, Sol De Jesus, Harini Sarva, Laura Y. Cabrera

**Affiliations:** 1 The Pennsylvania State University, University Park, PA, United States; 2 Baylor College of Medicine, Houston, TX, United States; 3 Penn State College of Medicine, Hershey, PA, United States; 4 Weill Cornell Medicine, New York, NY, United States

**Keywords:** clinicians perspectives, patient decision aid, decision making, deep brain stimulation, Parkinson disease

## Abstract

**Introduction:**

For people with Parkinson’s disease (PD), deciding whether to pursue deep brain stimulation (DBS) has become increasingly complex. Evidence suggests current approaches to collaborative decision-making may fall short of accepted standards. Thus, a decision support intervention, such as a patient decision aid (PtDA) may be warranted. PtDAs have been shown to improve patients’ knowledge, expectations, and participation in decision-making for other, similar healthcare decisions. We therefore sought to assess neurologists’ awareness of, and experience using, PtDAs, and to solicit their opinions on the ideal features of a PtDA for PD patients considering DBS.

**Methods:**

Sixteen United States-based neurology clinicians were interviewed about their experiences in treating and counseling PD patients considering DBS. These semi-structured interviews were analyzed using qualitative content analysis.

**Results:**

Seven general neurologists, eight movement disorders specialists, and one registered nurse in a movement practice participated in interviews. None of the clinicians had experience using a patient decision aid, and many were unfamiliar with the concept altogether. All largely expressed optimism about the potential utility of a PtDA for PD patients considering DBS. There was less consensus about the ideal format, content, and implementation of such a PtDA.

**Conclusion:**

Healthcare providers recognize the potential benefits of a PtDA to engage PD patients considering DBS. However, input from other stakeholders—particularly the patients themselves—is essential to validate these findings.

## Introduction

Of all the treatment decisions people with Parkinson’s disease (PD) face, deciding whether to transition from a standard pharmacologic treatment regimen to deep brain stimulation (DBS) can be particularly complex. This complexity stems from three main sources: disease-related uncertainty, factors influencing patient and clinician decisions, and technological and systems considerations.

Disease-related uncertainties in PD that contribute to decisional complexity include inter-patient symptom heterogeneity and a progressive course that is not always predictable. The shift towards considering DBS earlier in the patient’s disease progression has also raised novel questions about how stimulation may influence the long-term trajectory of the disease (Eijkholt et al., 2017; Hacker et al., 2020; Gilbert and Lancelot, 2021). A second source of complexity stems from the considerations of clinicians and patients involved in the decision-making. Patients, for example, vary in their values and preferences (Montemayor et al., 2022). Clinicians, similarly, must decide not only when or if the patient is a candidate for surgery but when to begin discussions about DBS and alternative therapies. Additionally, patients may have to consider not only *whether* to undergo DBS but also *when* might be the best time. The third source of decisional complexity comes from technological and systems considerations, including the expanding set of options available with the ongoing evolution of DBS technology and therapeutic landscape. Furthermore, clinicians in the United States. must make recommendations and decisions without up-to-date guidelines from professional organizations, leading to variability in practices across centers, including with respect to the question of timing (Cabrera et al., 2021; Mahajan et al., 2021). The approval of additional advanced therapies will only increase this decisional complexity, particularly insofar as patients may be unaware of certain treatment options and may hold concerning misconceptions about those treatments or Parkinson’s itself (Lökk, 2011; Nijhuis et al., 2016).

Ultimately, the complexity of this decision leads to a practical question: what can be done to help PD patients make the best decision, one that is well-informed and aligned with their values and preferences? One potential resource is a patient decision aid (PtDA). PtDAs are evidence-based tools designed to help patients make a specific medical decision where there is no singular optimal treatment choice and where the patient’s preferences and values are central to the decision. Designed as adjuncts to clinical counseling, PtDAs provide evidence-based information on treatment options and associated benefits and harms, and can help patients consider which benefits and harms are most important to them (Joseph-Williams et al., 2013). PtDAs come in print (e.g., pamphlets or flyers) or electronic (e.g., websites or apps) formats, and may be used during or outside of clinic visits. Evidence suggests that PtDAs facilitate shared decision-making, the process by which patients and clinicians arrive at a treatment decision together after considering the patient’s clinical situation as well as their values, preferences, and goals: a recent Cochrane review concluded that PtDAs improved patients’ knowledge, risk perception accuracy, and clarity of personal values, supported patients’ active participation in decision-making, and improved observer-reported assessments of shared decision-making (Stacey et al., 2024).

While PtDAs have been used extensively in other fields of medicine, they are relatively uncommon in neurology. The A-to-Z Inventory of Patient Decision Aids, an online listing of publicly available PtDAs that meet internationally accepted criteria, does not include an English-language PtDA for any treatment decision related to PD (Patient Decision Aids Research Group, 2025). Similarly, no randomized controlled trials exist comparing a PtDA to usual care for any PD treatment decision; indeed, neurodegenerative diseases writ large are vastly underrepresented in such studies (Stacey et al., 2024). Despite this, multiple groups have noted that decisions about advanced treatments for PD are good candidates for PtDAs (Sokol et al., 2019; Tanguay et al., 2025) and the evidence of PtDA efficacy for treatment decisions in a variety of other conditions (Stacey et al., 2024) suggests optimism is warranted about the potential efficacy of a PtDA for DBS in PD. To address this unmet need, we have a funded project to develop a PtDA for DBS in PD specifically designed for use within the United States. healthcare system. In doing so, we sought input from healthcare providers on key aspects of the PtDA (including content, format, and timing of delivery) to support its integration into clinical care (Witteman et al., 2021). In this paper, we report results from interviews with neurologists, focusing on their understanding and use of PtDAs as well as their thoughts on the potential utility and ideal features of a PtDA for PD patients considering DBS.

## Methods

We conducted semi-structured interviews with 16 neurology providers (eight movement disorders specialists, seven general neurologists, and one registered nurse in a movement practice) from around the United States. The study was approved by the Pennsylvania State University Institutional Review Board as an exempt study not requiring written consent from participants. In this section, to the extent possible, we have followed the consolidated criteria for reporting qualitative research; the completed checklist is included as a supplemental file (Tong et al., 2007).

### Sampling and recruitment

Inclusion criteria for participants was as follows: United States-based healthcare providers who care for people with PD who do, or may in the future, have DBS. A purposeful sampling approach was chosen in order to ensure that participants had relevant experience treating patients with PD and counseling them about DBS as a potential treatment option (Palinkas et al., 2015). Initial recruitment efforts began with movement disorders specialists (MDS). Potential participants were identified through our research team’s professional organizations and networks (including the Parkinson’s Study Group and the American Academy of Neurology) as well as through authorship of peer reviewed literature on DBS for Parkinson’s disease. In our initial interviews with MDS neurologists, several participants suggested seeking input from healthcare providers who interact with PD patients at other stages of their disease progression. Based on this feedback, we expanded our criteria to include general neurologists who see patients with PD, as well as a registered nurse who works in an MDS practice. We contacted potential participants by email with an invitation to participate in the study and sent one follow-up email if we did not receive a response initially. When scheduling interviews, we provided participants with a consent document explaining the study and the interview process. Participants were provided an opportunity to ask questions before the interview began. A study team member conducted each interview over Zoom or by phone.

### Procedure

We performed semi-structured interviews to explore clinicians’ perception of decision-making roles, the role of patients’ values in decision-making, and decisional barriers and facilitators. Interviews lasted between 24 and 60 min, with an average duration of 44 min. Interview guides were developed from researchers’ prior experience developing PtDAs and knowledge of domains relevant to decision-making about DBS for PD. The interview guide can be found at https://doi.org/10.17605/OSF.IO/U5BEM. Interviews were performed by a senior researcher with significant qualitative research experience (LC) or a postdoctoral scholar (HK) under their supervision. Interviews were recorded, transcribed, anonymized, and uploaded to Dedoose, a cloud-based application designed for qualitative and mixed-methods data analysis. A directed approach to qualitative content analysis was chosen, as existing theory and research was available to inform the development of a codebook (Hsieh and Shannon, 2005). The codebook was drafted (by HK and LC) to capture key elements of shared decision-making and specific features of PD and DBS and was then revised iteratively in consultation with the study team until a consensus was reached. HK, LC, or an undergraduate research assistant coded each interview, which were then reviewed by another team member (either HK or LC). After review, the team discussed each transcript’s coding in a meeting and reached consensus on any discrepancies. During coding, in instances where data could not be captured by existing codes or subcodes the codebook was amended, a process consistent with a directed approach to content analysis (Hsieh and Shannon, 2005). Here, we focus on clinicians’ understanding and use of PtDAs as well as their thoughts on the potential utility and ideal features of a PtDA for PD patients considering DBS. In our results section, we present relevant transcript excerpts to highlight and illustrate key findings—with non-content words and expressions removed for readability.

## Results

### Participants

We recruited diverse participants across sex, geographic location, practice type, and race and ethnicity. See Table 1 for the characteristics of our study sample.

**Table 1:** Description of the Study Sample

Demographic variable	Category	Frequency	Percentage
Gender	Male	7	43.75%
	Female	9	56.25%
Age	40-45	4	25.00%
	46-50	6	37.50%
	51-55	3	18.75%
	56+	2	12.50%
	Preferred not to answer	1	6.25%
Provider type	General neurologist	7	43.75%
	Movement disorders specialist	8	50.00%
	Registered nurse	1	6.25%
Years of clinical experience	<10	4	25.00%
	10-20	9	56.25%
	21-30	3	18.75%
Race/Ethnicity	White/Non-hispanic	8	50.00%
	Hispanic	2	12.50%
	Asian/Indian	3	19.75%
	Asian/Chinese	2	12.50%
	Black	1	6.25%
Facility/practice type	Academic medical center	7	43.75%
	Private practice	4	25.00%
	University-affiliated	3	18.75%
	Non-profit hospital	1	6.25%
	Community hospital	1	6.25%

### Familiarity with and prior use of PtDAs

When asked whether they were familiar with PtDAs or had used them in their practice, more than half either explicitly stated they were not familiar with PtDAs or indicated their lack of familiarity through follow-up questions. The remainder expressed some degree of familiarity with PtDAs but noted they had little or no experience using them in their practice. Follow-up discussions indicated that these clinicians had limited familiarity with PtDAs and often misinterpreted their purpose, viewing them as algorithms to guide clinician decision-making rather than tools intended to support patient decision-making. After the purpose of PtDAs was clarified, clinicians generally endorsed the utility of PtDAs for PD patients considering DBS but offered differing opinions on the ideal features of such a PtDA. See Table 2 for illustrative quotations of clinicians’ familiarity with PtDAs, as well as their views on the features outlined below.

**Table 2:** “At-a-glance” illustrative quotations across interview domains.

Interview domain	Example quotations
Familiarity with PtDAs	*CL06 (MDS)*: You mean like algorithms or electronic means of decision-making? *CL13 (GN)*: I have not overtly used them. I have kind of used the structure of some of them with, say, epilepsy or neuromuscular issues—of when or what treatments to consider. But I do not have a particular structured handbook that I follow.
Views on format	*CL03 (MDS)*: I think a flyer is not bad. I can always give them a flyer […] if it was like a questionnaire that I could just drop into their discharge paperwork for them to fill out that could help me focus my conversation. *CL06 (MDS)*: I would say, a web based; not just paper and pencil. Something that would have some sort of a graph […] like something with visual aids. *CL11 (GN)*: Younger patients, they love videos. But the patients over 65 prefer to read articles. *CL15 (GN)*: Maybe in bigger cities it’s different, but the patients I see struggle to access online resources. If it’s not with the help of a relative it might be difficult. So I think it could be a mix of both: you can have some online tools and then the same tool in a paper version to do in the office while the patient is waiting for the doctor.
Views on informational content	*CL09 (MDS)*: Lots of information in lay terms: the idea of the therapy itself, what to expect from it, the duration of effect, etc. *CL02 (MDS)*: I think you have to really pinpoint their major symptoms that they want to address, because sometimes they want everything to be resolved, which is not feasible.
Views on timing of introduction	*CL08 (RN)*: I would say in the regular return appointments with the movement disorder provider, because that’s when discussions about non-pharmacological treatments usually happen: that would be a good time to hand something like that out, or send it out electronically. *CL09 (MDS)*: I would want that decision aid to be in the hands of general neurologists who could give it to a patient before they see a movement disorder doc. Would that be the best place to provide that? Maybe. Yeah. And if not, then certainly when they see a movement disorder physician, then it should be provided. *CL05 (MDS)*: Whenever we start seeing some signs of progression from a motor standpoint, and we start making adjustments [to medication], I think that would be the time to start educating the person about DBS.
Views on PtDA standardization	*CL05 (MDS)*: I can see two separate ones: one for somebody who is a little earlier into the process, and another one who is like right at the gate of receiving the therapy. *CL09 (MDS)*: Patients at different stages need a different amount of information. So maybe if you really want to capture everybody it should be in the same aid, but with different sections, perhaps for different stages. […] Because if you have it in one aid, then then it reinforces the idea that it’s a progressive disease, and that’s a good thing, because it helps patients envision themselves at future stages.

PtDA (patient decision aid), CL (clinician), MDS (movement disorders specialist), GN (general neurologist), RN (registered nurse).

### Views on format

There were no strong preferences regarding how decision aids should be formatted, though several clinicians endorsed a web-based PtDA for its ability to include images, graphics, and/or videos. Other clinicians were more equivocal, noting that patients vary in their preferences and technological competencies.

### Views on informational content

Clinicians most frequently said that the PtDA should provide basic information about DBS and the steps involved in having DBS placed. Clinicians also suggested including information about the pros and cons (or benefits and risks) of DBS, symptoms DBS can and cannot address, candidacy criteria, and the potential effects of DBS on patients’ medication regimen. Only one clinician mentioned including information about other treatment options. Clinicians also indicated a desire for the PtDA to help address patients’ fears about DBS, be written in simple, concise language, and answer frequently asked questions.

### Views on timing of introduction

A majority of clinicians identified the ideal timing for introducing patients to the PD-DBS PtDA at a point relative to the patient’s MDS clinic visits, often suggesting the first MDS clinic visits or even before. Other clinicians suggested introducing the PtDA once patient symptoms progress despite medical management. The remaining few clinicians referred simply to “later” in the patient’s disease progression, so as not to overwhelm patients with too much information too soon or “earlier” because PD patients were being referred to movement specialists for DBS consultation too late or because earlier knowledge of DBS might influence patients’ decisions later in the disease. The two clinicians recommending earlier introduction also suggested that it would be helpful if patients revisited the PtDA periodically throughout the course of their disease.

### Views on clinician involvement

With respect to which type of clinician, if any, should be involved in administering the PtDA, general neurologists almost unanimously recommended that a general neurologist, primary care physician (PCP), or an advanced practice provider or registered nurse (RN) in their practice should be involved. MDS neurologists offered more varied responses: two suggested a MDS or DBS specialist, one suggested a general neurologist or an advanced practice provider or RN in their practice, one asserted that patients should complete the PtDA on their own because clinicians would not have time, and two suggested that the design of the PtDA would determine whether a clinician should be involved.

### Views on PtDA standardization

There was no consensus on whether a single PtDA could accommodate all the different time points at which patients might encounter the decision about DBS. That said, there was broad agreement that patients who are earlier versus later in their disease progression might well have different concerns.

When asked whether any sociodemographic differences among PD patients would warrant a separate PtDA, clinicians reported that differences in cultural values, educational background, health literacy, and/or current knowledge of PD and DBS should be accounted for. However, clinicians were largely noncommittal as to whether those differences warrant multiple PtDAs.

## Discussion

Overall, our results suggest that even though neurologists are not familiar with PtDAs they still recognize the potential of a PtDA to facilitate shared decision-making with PD patients who might be candidates for DBS. Moreover, while there was less agreement about the ideal features of a DBS-PD PtDA, their recommednations regarding format, informational content, timing of introduction, and clinician involvement are informative for developing and testing of such a PtDA.

### Clinicians lacked familiarity with PtDAs but recognized their potential

We found that both MDS and general neurologists were unfamiliar with PtDAs. This finding is unsurprising, as there are few mechanisms through which a neurologist might learn about PtDAs in the ordinary course of practice. Moreover, there is little discussion of PtDAs in clinical neurology journals, and few PtDAs exist for decisions about neurological conditions. The A-to-Z Inventory of Patient Decision Aids lists only 22 PtDAs for 8 different neurological conditions, including Alzheimer’s (*n* = 3), ALS (*n* = 3), and dementia (*n* = 8) (14). By comparison, PtDAs are much more common in oncology, with the A-to-Z Inventory listing 42 PtDAs across 11 different cancer types. The only PtDA for a PD treatment decision included in the inventory addresses levodopa-carbidopa intestinal gel (LCIG) and is available only in French. We are also aware of two additional PtDAs for advanced PD treatments that are not included in the A-to-Z Inventory. One, developed by a research team in the Netherlands, is available only in Dutch (Nijhuis et al., 2025). The other, which came to our attention after starting our study, is being developed by a team at the University of Colorado whose research focuses on the differing decisional needs of women and men (Fullard et al., 2026). By contrast, our tool is being designed with a focus on the timing of the DBS decision relative to the patient’s disease stage. Additionally, we incorporated stakeholder input from clinicians, PD patients, and care partners from across multiple United States states, which we hope will broaden the applicability and reach of our developed PtDA.

Despite their lack of familiarity with PtDAs, participants were optimistic about the potential utility of a DBS-PD PtDA. However, there was less agreement on the ideal format, content, timing of introduction, clinician involvement, and whether a single PtDA could account for the heterogeneity of PD patients and PD itself.

### Features of DBS-PD PtDA

Clinicians did not express a clear preference for a web-based or paper-based PtDA. This ambivalence is reflected in the data on extant PtDAs: in their review, Stacey et al. (2024) found that 43% were paper, 33% were web-based or a computer program, and 16% were a combination of the two. Additionally, the only PD PtDA included in the A-to-Z Inventory was developed as both a paper handout and an electronic booklet (Tanguay et al., 2025). There is some evidence that web-based PtDAs are more likely to be integrated into clinical practice, but the characteristics and preferences of the patient population in question must be taken into account.

Overall, the information clinicians recommended for inclusion in a DBS-PD PtDA largely aligns with the International Patient Decision Aid Standards (IPDAS), a framework used to guide the development and evaluation of PtDAs. According to IPDAS criteria, PtDAs must describe the health condition, list options, describe the positive and negative features of those options, and either ask patients to consider which of those positive and negative features matter most to them or describe what it’s like to experience the consequences of those options (Stacey et al., 2025). However, no clinician mentioned all of these informational components. A noteworthy discrepancy between IPDAS criteria and our findings is that only one clinician mentioned including information about alternative device-aided and/or surgical therapies, such as LCIG, continuous subcutaneous apomorphine infusion (CSAI), and focused ultrasound (FUS). This omission of alternative therapies from most participants’ suggestions for the informational content of the PtDA is relevant as empirical data suggests that PD patients often lack information about these alternatives, with some authors arguing that this data is indicative of shortcomings in current counseling practice (Lökk, 2011; Nijhuis et al., 2019; Tanguay et al., 2025). IPDAS guidelines state that PtDAs should provide balanced information on all reasonable treatment options (Martin et al., 2021). However, the question of which of those other therapies present viable alternatives to DBS may not have a straightforward answer. One general neurologist, for example, expressed the opinion that while other device-aided and/or surgical therapies for PD exist, none are comparable to DBS. If this view is commonly held, it would explain why clinicians in our study did not mention alternative therapies that should be included in the PtDA. Another potential explanation, seen in our interviews with general neurologists and reflected in other research, is that they did not know of any other device-aided and/or surgical treatments for PD (Nijhuis et al., 2016). While this would not explain why the MDS clinicians did not mention alternative treatments, other contextual factors may influence clinicians’ perceptions of DBS alternatives, including the availability of these therapies, patient preferences, and clinician experience.

### Perspectives on implementation

Most clinicians, particularly the movement specialists, indicated that the ideal time to introduce the PtDA should be determined with reference to the patient’s initial visits with an MDS provider. However, this is a somewhat problematic referent, as a broad-based 2023 study found that only 9% of PD patients saw a movement specialist. Further, that same study found that of the remaining 91%, only 51% saw a neurologist, meaning that the remaining 40% of people with PD only saw their PCP or saw no physician at all that year (Pearson et al., 2023). Additionally, even when patients are referred to movement specialists, that referral may come after the patient has passed the ‘ideal window’ for DBS, a view articulated by several clinicians in our study that comports with previous research (Cabrera et al., 2021). Thus, it may be the case that the ideal implementation setting for the PtDA is in visits with a general neurologist or a PCP.

While PtDAs are generally designed for patients to use independently, evidence suggests that clinician involvement is positively correlated with both implementation and a broader range of patient benefits (Joseph-Williams et al., 2020). As such, it is encouraging that most of the clinicians we interviewed recommended that one or more healthcare providers be involved. Some clinicians noted that although engaging with the PtDA would be worthwhile, healthcare teams often lack the time to do so and may struggle to financially justify that use of time. But data show that consultations are only 1.5 min longer when clinicians use a decision aid during encounters with patients (Stacey et al., 2024). Additionally, PtDAs have been shown to improve clinicians’ experiences with decision-making, and those who used them as part of clinical trials often expressed the desire to continue doing so after the trial ended (Stacey et al., 2024; Tanguay et al., 2025).

Another consideration for implementation is whether multiple decision aids are needed for different stages of PD or sociodemographic considerations. This question is not one that has been discussed in the literature, and considering that clinicians in our study expressed differing opinions on this question, more research is needed to explore the benefits and drawbacks of such stratification of a DBS-PD PtDA. For now, the focus should be on facilitating the clinical implementation of a single, well-designed PtDA: despite the proven efficacy of PtDAs, their implementation in real-world healthcare decision-making remains a significant challenge for the field (Joseph-Williams et al., 2020; Stacey et al., 2024). In a survey of PtDA researchers, the most commonly cited barriers to PtDA implementation included a lack of funding or infrastructure support, outdated PtDAs, and a lack of delivery mechanisms (Stacey et al., 2024). Common facilitators, on the other hand, included online delivery, end-user involvement in development, endorsement by organizations or clinical practice guidelines, and clinician awareness (Stacey et al., 2024). Promoting neurology clinicians’ awareness of, and familiarity with, PtDAs is thus an important component in the eventual successful deployment of a DBS-PD PtDA. While our participants were not familiar with PtDAs, some of them *were* aware of treatment decision algorithms designed for *clinicians*, such as the MANAGE-PD tool. Despite the fact that these tools have different goals, familiarity with these decision algorithms might facilitate understanding of PtDAs and the incorporation of a PtDA into clinical practice (Antonini et al., 2021).

## Limitations

A major limitation of our study, as with most qualitative studies, is that the relatively small sample of interviews may lessen the generalizability of our findings, although our chosen sample size falls within accepted norms for this type of research. Relatedly, the semi-structured nature of the interviews allows for variability in the flow of conversation and the opportunity for respondent and interviewer bias to influence findings. Furthermore, while we aimed to recruit a demographically diverse group of MDS and general neurologists, we are unable to assess the extent to which our sample reflects the national demographics of those providers as, to our knowledge, those data are not publicly available. Our study sample focused on MDS and general neurologists because of the frequency of their interactions with patients with PD and because of their involvement in counseling those patients about DBS. That said, the present paper does not include the perspectives of neurosurgeons, neuropsychologists, or other healthcare providers who may be involved in assessing patients for DBS candidacy. Neither does it include the perspectives of primary care physicians who, as noted above, often care for patients with PD, and who might utilize a PtDA in collaboration with those patients as they consider referral to a MDS neurologist for DBS candidacy evaluation. However, input from these other providers will be pursued as the PtDA development process continues: not only can they provide valuable feedback on the acceptability of the PtDA (O’Connor and Cranney, 2002) but they will also be important partners in implementation.

Additionally, clinicians’ lack of familiarity with PtDAs (including misunderstanding them to be algorithms to help clinicians decide which treatment to recommend to patients) may have influenced the study findings. While we described PtDAs before soliciting feedback on a hypothetical PtDA for PD patients considering DBS, a deeper understanding and more time to reflect on features of PtDAs might have yielded different responses. Even so, as our participants are experts in counseling PD patients through treatment decisions, their insights and suggestions still reflect that expertise and provide a valuable starting point for the development of a prototype PtDA about DBS for PD. Further refinement will occur through additional rounds of interviews.

Lastly, our study’s focus was on the United States healthcare system, a decision made because of the variability in how DBS is utilized for PD in the United States (e.g., in referral pathways, providers involved in patient care and decision-making, candidacy assessment procedures, and device availability). Trying to incorporate the complexities and nuances of other healthcare systems would further complicate the PtDA development process, perhaps to the detriment of the resulting PtDA. Additionally, our study team consists entirely of United States-based researchers with little experience in the healthcare systems of other countries. We note the efforts of other research teams engaged in the development of PtDAs for advanced PD treatments abroad, and encourage others to join in this important work (Nijhuis et al., 2025; Tanguay et al., 2025).

## Conclusion and future directions

We found that while neurology clinicians are not familiar with PtDAs, they are optimistic about the value of a PtDA for PD patients considering DBS. Their insights reflect a wealth of experience treating patients with PD and their suggestions provided a strong foundation from which to begin development of our PtDA, in concert with input from patients and care partners. The perspectives of these latter stakeholders will be reported in future manuscripts, along with additional insights from clinicians. Where clinicians’ responses were largely consistent, as was the case with many of the suggestions about the informational content of the PtDA, those suggestions were incorporated into initial iterations of the prototype. Even where clinicians’ suggestions were more variable (e.g., which, if any, alternative treatments should be included) that ambiguity informed the initial design of the prototype as well as the creation of survey intstruments to assess its acceptability (O’Connor and Cranney, 2002). Additional stakeholder input on those remaining questions about the content and features of the DBS-PD PtDA will be sought in future interviews as the development process continues.
